# Promising biomarkers for mild cognitive impairment and Alzheimer’s disease: Levels of soluble endothelial glycocalyx products

**DOI:** 10.4103/NRR.NRR-D-24-01033

**Published:** 2026-01-27

**Authors:** Carmela Rita Balistreri, Roberto Monastero

**Affiliations:** Cellular, Molecular and Clinical Pathological Laboratory, Department of Biomedicine, Neuroscience and Advanced Diagnostics (Bi.N.D.), University of Palermo, Palermo, Italy; Memory and Parkinson’s disease Center, Policlinico “Paolo Giaccone”, Palermo, Italy; Department of Biomedicine, Neuroscience and Advanced Diagnostics (Bi.N.D.), University of Palermo, Palermo, Italy

A previous study has described a strong relationship between cardio- and cerebrovascular risk factors and the onset and progression of cognitive impairment (CI), and Alzheimer’s disease (AD), although their pathophysiology remains not completely understood (Balistreri, 2021). Consequently, our group has recently described the crucial role of the endothelium dysfunction, as well as of its dysfunctional endothelial glycocalyx (eGCX) and the related cellular and molecular mechanisms, in the onset of neurological pathologies, including stroke and AD (Balistreri et al., 2024; Balistreri and Monastero, 2025). The endothelium is not only the essential element of the cardiovascular system, but it also constitutes the essential component of any tissue in the human body, including the neurovascular unit and the brain–blood barrier (Balistreri and Monastero, 2023). In this case, their dysfunction triggers brain inflammation and the consequent pathological mechanisms linked to the onset and progression of CI and AD (Balistreri and Monastero, 2023). Therefore, the impact of eGCX dysfunction in the onset and progression of these diseases has also become of growing interest. The dysfunction of eGCX is characterized by its degradation, as shown by the high systemic levels of eGCX shedding products also described in AD (Balistreri et al., 2024; Balistreri and Monastero, 2025). However, data on this topic are still limited and mainly based on pre-clinical studies. Therefore, in this pilot study, we assessed whether the degradation of eGCX and endothelial dysfunction characterize AD, and in its prodromal phase, the so-called mild cognitive impairment (MCI). For this purpose, we quantified the systemic levels of certain eGCX degradation products and other molecules related to both eGCX and endothelium dysfunction, including Elabela-54, -21, and -32, syndecans (SDC) 1-4, thrombomodulin (CD141), and vascular endothelial growth factor (VEGF) in subjects with MCI and AD compared with healthy controls (HC).

**Methods:** A number of 60 subjects, 20 MCI cases (men age of 74.6 ± 5.8 years, age range 60–82 years; 12 males and 8 females), 20 AD cases (mean age 72.4 ± 8.1 years, age range 69–85 years; 8 males and 12 females), and 20 matched controls for age and sex (mean age 73 ± 1.4 years, age range 66–83 years; 10 males and 10 females), were included in the present study. Dementia was diagnosed according to the Diagnostic and Statistical Manual of Mental Disorders (DSM-5, American Psychiatric Association, 2013) as major neurocognitive disorder and probable AD according to National Institute on Aging and Alzheimer’s Association criteria (Bieger et al., 2024). Exclusion criteria were dementia due to other general medical conditions (e.g., head trauma, severe metabolic or endocrine disease, brain tumor, and stroke), a relevant history of alcohol or substance abuse or dependence, delirium, and psychiatric disorders preceding the onset of dementia. MCI has been classified according to the modified Petersen criteria (Winblad et al., 2004). Subjects with MCI and AD included in the study were assessed with a multidimensional protocol that included: demographic characteristics, comorbidities, standardized neurological examination, neuropsychological and behavioral examination, screening blood tests for dementia. Additionally, MCI and AD performed routine MRI at 1.5T to rule out relevant vascular lesions, which could affect cognition. Controls were individuals with normal global cognition (Montreal Cognitive Assessment, adjusted scores ≥ 24) (Siciliano et al., 2019) and normal functioning in instrumental activities of daily living. Venous blood samples were collected from all enrolled subjects in a fasting state (> 8 hours without food administration). Plasma samples were obtained immediately after collection by centrifuging whole blood (3500 rpm at 4°C for 10 minutes) and then stored at –80°C for further analysis. Plasma levels of ELA-54, -32, and -21 were determined using a commercialized human Enzyme-Linked Immunosorbent Assay kit (Peninsula Laboratories International, Inc., USA). Plasma levels of SDC-1, -2, -3, and -4 were detected by using Enzyme-Linked Immunosorbent Assay commercial kits (SDC 1: Bio-Techne Ltd., Abingdon, UK; SDC 2: My BioSource, San Diego, USA; SDC 3 e SDC 4: R&D Systems, Minneapolis, MN, US). Furthermore, thrombomodulin (CD141) and VEGF were quantified by using Enzyme-Linked Immunosorbent Assay commercial kits (Bio-Techne Ltd., Abingdon, UK). The operating procedures for all molecules evaluated were carried out according to the manufacturer’s instructions. Concerning statistics, normal distribution and homogeneity of variables were tested with Kolmogorov-Smirnov and Levene’s test, respectively. Mean values with standard deviations were calculated for quantitative variables due to a normal distribution. Differences between normal variables were compared by one-way analysis of variance (ANOVA) with Scheffe’s *post hoc* test for multiple comparisons. Due to the multiple comparisons, the Bonferroni-corrected significance threshold was set at *P* = 0.0125 (0.05/4) to reduce the possibility of type I errors. For all analyses, the significance level was set at *P* ≤ 0.05.

**Figure 1:**
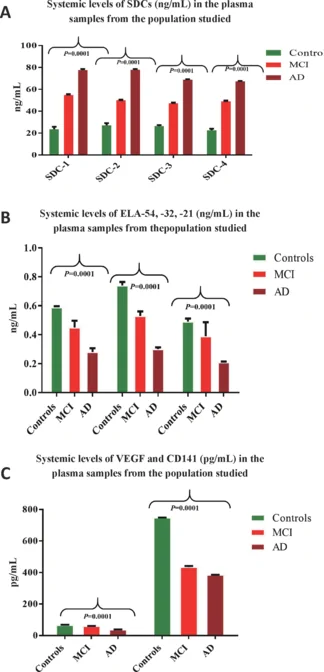
Representation of the systemic levels in the population studied. (A) Systemic levels of ELA-54 peptide and its degradation products, reporting the significant *P* values detected by one-way analysis of variance (ANOVA) test corrected by Bonferroni; (B) systemic levels of the four SDCs, reporting the significant P values detected by ANOVA test corrected by Bonferroni; (C) systemic levels of vascular endothelial growth factor (VEGF) and CD141, reporting the significant *P* values detected by ANOVA test corrected by Bonferroni (for further details it postpones to the results’ section of article).

**Results: *Demographic and the detected biochemical parameters displayed in Table 1:*** Significant differences were observed by comparing the plasma levels of the detected molecules among the three diverse groups, as shown in **Table 1**. Precisely, ANOVA revealed significant within-group differences in ELA-54 peptide and its degradation products, ELA-32 and -21, with decreasing values of these molecules moving from healthy controls (higher values) to MCI (intermediate values) and AD (lower values) (ANOVA *P* ≤ 0.0001 for all comparisons; **Figure 1A**). Additionally, after Sheffè’s *post hoc* test, control subjects had significantly higher plasma levels of the 3 ELA molecules than MCI and AD, with MCI in turn having significantly higher values than AD (Shefè *post hoc*, *P* ≤ 0.0001 for all comparisons). Therefore, significantly decreasing expression kinetics of the peptides described above characterized the cases than the controls, and particularly AD subjects. Regarding the circulating levels of the four SDC molecules, an opposite trend with respect to that found for ELA was observed. Specifically, control subjects (both after ANOVA and after pair-comparison with Sheffè’s *post hoc* test) presented the lowest values for each SDC evaluated, with intermediate values presented by MCIs and higher values by ADs, and all these differences were significant (*P* ≤ 0.0001 for all comparisons; **Figure 1B**). Finally, we also observed a significant decreasing trend moving from controls, MCI, and AD in what concerns thrombomodulin (CD141) and VEGF levels (*P* ≤ 0.0001 for all comparisons after Shefè *post-hoc* analysis).

**Table 1:** Demographic and biochemical parameters in our study population

Variable	HC	MCI	AD	*P*-value ANOVA	*Post hoc*
Age	74.0±6.7	74.7±6.7	72.4±6.3	ns	ns
Ela54	0.59±0.007	0.45±0.047	0.28±0.026	0.0001	HC>MCI>AD
Ela32	0.74±0.025	0.53±0.031	0.30±0.013	0.0001	HC>MCI>AD
Ela21	0.49±0.022	0.39±0.096	0.21±0.005	0.0001	HC>MCI>AD
SDC1	23.6±2.19	77.6±0.94	54.8±0.55	0.0001	MCI>AD>HC
SDC2	27.0±2.11	77.9±0.67	50.0±0.39	0.0001	MCI>AD>HC
SDC3	26.4±0.99	68.8±0.52	47.2±0.52	0.0001	MCI>AD>HC
SDC4	22.6±1.47	67.2±0.49	49.0±0.51	0.0001	MCI>AD>HC
VEGF	66.6±1.81	38.2±0.49	60.5±0.67	0.0001	HC>AD>MCI
CD141	750.4±5.42	385.4±0.81	435.2±6.17	0.0001	HC>AD>MCI

Data are expressed as the mean ± SD. *P* values were assessed by one-way analysis of variance (ANOVA) with Scheffe’s post hoc test for multiple comparisons. Due to the multiple comparisons, the Bonferroni-corrected significance threshold was set at *P* = 0.0125 (0.05/4) to reduce the possibility of type I errors. For all analyses, significance level was set at *P* ≤ 0.05. AD: Alzheimer's disease; HC: healthy controls; MCI: mild cognitive impairment; ns: not significant.

**Discussion and conclusions:** Several pathological conditions have been recently related to eGCX and endothelium dysfunction, including neuroinflammation and neurodegeneration (Balistreri and Monastero, 2023, 2025; Balistreri et al., 2024). These latter conditions are, in turn, closely linked to the development and progression of neurological diseases, such as AD (Balistreri and Monastero, 2023, 2025; Balistreri et al., 2024). This evidence is supported by studies, emphasizing the key role of eGCX in the activation of several pathways, including the ELA/APJ pathway, which contributes to the maintenance of endothelial integrity and its permeability, influencing nitric oxide production (Monastero et al., 2023). eGCX and endothelium dysfunction also contributes evoking neuroinflammation, as mentioned above, which plays a critical role in the progression of AD (Balistreri, 2021; Monastero et al., 2023; Balistreri et al., 2024; Hroudová and Fišar, 2024; Balistreri and Monastero, 2025). Consistent with this evidence, a significant reduction in circulating levels of the three ELA molecules was observed in subjects with MCI and AD compared to controls in the present study. Subjects with AD had values more than halved compared to controls. Mounting evidence reports, indeed, that the ELA/APJ pathway is a prominent player in the pathogenesis of different neuronal and mental diseases, such as MCI and AD, among others (widely quoted in Monastero et al., 2023).

Similarly, but with an inverse trend, we found a significant increase in the levels of SDC 1–4 going from controls to MCI and AD. In particular, the latter had blood values more than double those of the controls. This finding agrees with the recent evidence reported by Hudák et al. (2022), who described an increase in SDC-3 in ADs compared to controls. These authors described that SDC3 plays a key role in facilitating the endocytosis of amyloid-beta (Aβ) 1–42 and its fibrillation, thus contributing to the progression of the cerebral amyloidosis process in individuals with AD (Hudák et al., 2022). Furthermore, it has been shown that the increase in Aβ polymerisation is mediated by the interaction of ApoE (the main susceptibility gene for sporadic AD) with SDC-3 (widely quoted in Balistreri et al., 2024), and this would contribute to the induction of neuroinflammation. *In vitro* studies have also reported that inflammatory cytokines, such as TNF-α, mediate the release of SDC-3, so this molecule would play a multiple role in contributing to the amyloidogenic and neuroinflammatory processes (Balistreri and Monastero, 2023, 2025). Confirming the latter hypothesis, in a transgenic mouse model of AD, peripheral monocytes showed an amplified expression of SDC-3, which significantly correlated with brain amyloid burden (Hudák et al., 2022). Therefore, systemic levels of SDC3 could be used as a possible biomarker for early diagnosis and progression of the neurodegenerative process underlying AD.

Finally, in the present study, we found that subjects with MCI and particularly those with AD had significantly lower levels of VEGF and CD141 than controls. This datum is interesting, even if the recent evidence is contrasting. A recent meta-analysis including 24 studies reports that VEGF is more effective for the diagnosis of vascular dementia (VD), but vascular factors are also an important contributor in AD. Thus, further studies are necessary (Xu et al., 2024). In contrast, another meta-analysis, better structured, underlines a significant association of some biomarkers of endothelial dysfunction in AD, such as CD141, whereas VD presented mostly an involvement of coagulation cascade components (Loures et al., 2019).

Overall, the preliminary data described in this report confirm that disruption of the endothelium (and related molecules) is a phenomenon that occurs early in subjects with AD, being detectable already in its prodromal phases (i.e., MCI). Accordingly, this datum is agreement with the results evidenced by a Hispanic group of researchers. They have recently evidenced in a meta-analysis conducted in 32 studies, including 485 patients with mild cognitive impairment, 568 with VD, 1781 with AD, and 2855 participants without dementia, that patients with AD had elevated levels of a series of biomarkers of endothelial dysfunction (i.e., von Willebrand factor, D-dimer, plasminogen activator inhibitor-1, thrombomodulin, and homocysteine) compared to controls. Differently, patients with VD had a significant increase in some factors related to the coagulation cascade (Loures et al., 2019). In 2024, another group has also suggested the importance of assessing the endothelium dysfunction of brain-blood barrier and its association with AD, cognitive impairment, and neuroinflammation (widely quoted in Balistreri et al., 2024).

In conclusion, the data of this preliminary report suggest that the presence of endothelial disruption is a pathophysiological process that occurs early during the AD-related neurodegenerative process. These results seem quite promising but further studies, conducted in a large series of subjects with MCI and AD, are warranted to confirm them. If confirmed, molecules related to endothelial dysfunction will then be validated as possible diagnostic biomarkers for AD.

*This work was supported by grants from the Next Generation EU - MUR D.M. 737/2021 - Project PSEBPEHRD- CUP B79J21038330001 (to CRB).*
